# Use of Chloride Ot Sodium in Diseases of the Eye

**Published:** 1844-08-24

**Authors:** 


USE OF CHLORIDE OT SODIUM IN DISEASES OF THE EYE.

Dr. Tavignot (L'Experience) employs the chloride of sodium, as a topical application, with advantage, in the treatment of different forms of inflammation of the eye, and more particularly in ulcerations of the cornea, in the form of ointment or collyrium, and sometimes even in substance. He considers it to be more efficacious than nitrate of silver, and other substances which are commonly applied, in such cases, and less likely to produce permanent irritation, or to act as an escharotic. The ointment is prepared in the proportion of one to four drachms of common salt to the ounce of lard; the weakest preparation being used at the commencement of the treatment, and the collyrium is made with from one to three drachms to the ounce of water. One drachm to the ounce will be sufficiently strong for ordinary cases.—*Ibid.*

